# Roles of Retinoids and Retinoic Acid Receptors in the Regulation of Hematopoietic Stem Cell Self-Renewal and Differentiation

**DOI:** 10.1155/2007/87934

**Published:** 2007-08-06

**Authors:** Louise E. Purton

**Affiliations:** Center for Regenerative Medicine, Massachusetts General Hospital, Harvard Medical School, Harvard Stem Cell Institute, Boston, MA 02114, USA

## Abstract

Multipotent hematopoietic stem cells (HSCs) sustain blood cell production throughout an individual's lifespan through complex processes ultimately leading to fates of self-renewal, differentiation or cell death decisions. A fine balance between these decisions in vivo allows for the size of the HSC pool to be maintained. While many key factors involved in regulating HSC/progenitor cell differentiation and cell death are known, the critical regulators of HSC self-renewal are largely unknown. In recent years, however, a number of studies describing methods of increasing or decreasing the numbers of HSCs in a given population have emerged. Of major interest here are the emerging roles of retinoids in the regulation of HSCs.

## 1. INTRODUCTION

### 1.1. Cell fate decisions of HSCs

**Figure 1:**
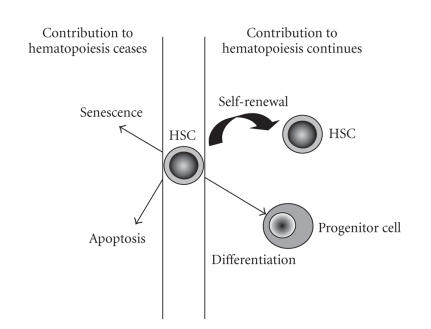
Hematopoietic stem cells (HSCs) undergo fate decisions including self-renewal (resulting in the production of more HSCs), differentiation (producing more mature progenitor cells), senescence, or apoptosis, the latter two resulting in cell death.

HSCs maintain hematopoiesis through fine processes involving cell self-renewal, differentiation, or death decisions (Figure 1). A balance between these choices is required for homeostasis of the blood cell system. Imbalances can result in severe consequences to the health of the individual: bone marrow failure can result from reduced HSC self-renewal or enhanced cell differentiation or death, whereas bone marrow diseases such as leukemia largely result from inhibition of cell differentiation or death of a progenitor cell in addition to enhanced self-renewal of the leukemia-initiating cell.

### 1.2. Functional self-renewal of HSCs

HSC self-renewal is defined in this review as the retention of the functional capacity of the HSC after cell division. Studies investigating the regulation of HSC self-renewal have predominantly focused on *ex vivo* culture systems, which, while allowing more direct examination of the roles of certain factors in the absence of others, may not provide information as to physiological regulators of HSC self-renewal, especially with regards to microenvironmental influences. The importance of the bone marrow microenvironment in regulating hematopoiesis has been demonstrated by the capacity of in vitro bone marrow stromal cell cultures to support hematopoietic stem cells [1]. To date, however, both in vitro and in vivo studies of the microenvironmental regulators of HSC self-renewal have been relatively few. This is due in part to the complexity of the multicellular stromal cell system in addition to the lack of identification of the HSC-regulatory cells within the stromal cell compartment, also known as the HSC niche. The recent identification that a key component of the in vivo HSC niche is the osteoblast, or bone-forming cells [2, 3], in addition to the observation that a change in the osteoblast niche size has a marked impact on the ability of the HSCs to self-renew, has now opened the field for further investigation in the context of the microenvironmental regulation of HSC self-renewal. The studies reported here have predominantly been performed independent of the HSC niche, thus represent intrinsic, or cell-autonomous, roles in HSC function.

### 1.3. Retinoids

Vitamin A (retinol) and its derivatives, collectively referred to as retinoids, are essential for normal development and homeostasis of vertebrates as shown by their profound effects as morphogens during embryonic development [4] and by their crucial role in the physiology of many organs. The mechanisms responsible for the diverse effects of retinoids have yet to be fully elucidated but are ultimately dependent on the specific binding of retinoid ligands to nuclear receptors, which as ligand-dependent transcription factors regulate complex programs of gene expression in various target cells and tissues. 

Retinol is not synthesized by animals, but is obtained from a variety of food sources in the form of carotenoids (from fruits and vegetables) or retinyl esters (from animal sources, especially liver). Retinol is a teratogenic agent: vitamin A deficiency results in multiple and severe developmental defects in many different organs. Paradoxically, excess retinol (hypervitaminosis A) causes many serious developmental defects. These findings not only highlight the importance of retinoids in regulating many developmental processes, but they also emphasize how critical it is to regulate retinoid levels within the body [5, 6].

Retinol is not biologically active, but is metabolized within the body by a series of enzymes into a range of biologically active forms of aldehyde or carboxylic acids [7]. The major aldehyde form, 11-*cis* retinal, is crucial for normal processes involved in vision. The major carboxylic acid form, all-*trans* retinoic acid (ATRA), is required for the regulation of gene transcription by vitamin A [7], and is the form of vitamin A that has roles in hematopoiesis.

### 1.4. Retinoic acid receptors

The biologic effects of ATRA and other retinoids are mediated by two families of receptors belonging to the nuclear hormone receptor superfamily: the retinoic acid receptor (RAR) and retinoid X receptor (RXR) families [8]. These receptors are encoded by a number of related genes, each of which generates distinct subtypes (designated *α*, *β*, and *γ*), and each subtype has at least 2 different isoforms generated by alternative splicing [9, 10]. The retinoid receptors are highly conserved between species and show complex stage- and tissue-specific patterns of expression, suggesting a molecular basis for the diverse biological effects of retinoids. RAR/RXR heterodimers are the functional units responsible for the transduction of retinoid signals [11], binding to specific retinoic acid response elements (RAREs) present in the promoters of their target genes to regulate transcription [8]. The retinoid receptors have two contrasting roles in the regulation of transcription. When not bound to ligand, the RAR/RXR heterodimers repress transcription. In contrast, in the liganded state, these receptors activate transcription. ATRA preferentially binds to RARs but not RXRs. RARs are specific to the retinoid signalling pathway, whereas RXRs also heterodimerize with other members of the nuclear hormone receptor superfamily. There are very few reports on the roles of the RXRs in hematopoiesis, however, a recent report showed that mice lacking RXR*α*, which is widely expressed by hematopoietic cells, have normal hematopoiesis in vivo [12]. 

### 1.5. The effects of retinoids on hematopoiesis

In hematopoiesis, the best documented action of retinoids is the induction of differentiation of primary leukemic blasts from patients with acute promyelocytic leukemia (APML), and therapies that include ATRA treatment achieve sustained remission in approximately 75% of patients [13].

Numerous studies investigating the effects of ATRA on normal human and murine hematopoiesis reached variable and often contrasting conclusions. Some reports suggested that ATRA enhanced the proliferation of human progenitor cells [14–16], whereas others demonstrated an inhibitory effect on both proliferation and differentiation of both human and murine progenitor cells [17–21]. It must be noted that inhibition of proliferation could be interpreted in different ways depending on the cell type: inhibition of proliferation in maturing cells is associated with cell cycle arrest accompanied by differentiation of the cell, as observed when immature granulocytes differentiate in response to ATRA [22]. In contrast, in the context of immature hematopoietic cells, especially HSCs, which are relatively quiescent cells [23], inhibition of proliferation (or more appropriately, slowing of proliferation) may be associated with maintenance of a primitive state of the cell. This has been observed when immature hematopoietic cells enriched for HSCs were cultured with ATRA [18, 24, 25].

**Figure 2:**
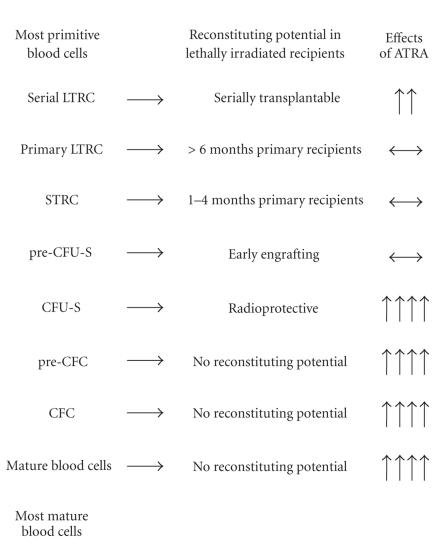
A summary of the effects of ATRA on HSC-containing LKS+ cells. LTRC = long-term repopulating HSCs, STRC = short-term repopulating HSCs, CFU-S = colony-forming unit-spleen, CFC = colony-forming cell. Upward pointing arrows indicate increase in potential, the number of arrows indicates the magnitude of increase, sideways arrows indicate maintenance of potential. Modified from [25].

Indeed, these contradictory effects of ATRA in hematopoiesis may be resolved by the recent finding that ATRA has pleiotropic effects on murine hematopoietic cells. In accord with its effects in APML, ATRA was found to be a potent inducer of terminal maturation of normal promyelocytes into granulocytes [24]. However, on more immature populations of hematopoietic cells enriched in hematopoietic stem and progenitor cells (lineage-negative, c-*kit* -positive, Sca-1-positive cells [LKS+]) [26], ATRA exhibited the opposite effect. The addition of ATRA to *ex vivo* liquid suspension media containing cytokines markedly prolonged and enhanced the production of colony-forming cells (CFCs) and colony-forming unit-spleen (CFU-S) and maintained pre-CFU-S production from cultured LKS+ [24, 25]. In addition, ATRA enhanced the maintenance of in vivo repopulating HSCs from this cultured cell population [25]. Additional studies demonstrated that ATRA enhanced the self-renewal of serially transplantable HSCs [27]. These effects of ATRA were restricted to a relatively primitive cell population: in contrast to that observed for LKS+ cells, lineage-negative, c-*kit*-positive, Sca-1-negative cells (LKS−), which exhibit CFU-S and CFC potential, but do not contain HSCs [26], differentiated in response to ATRA [24]. A summary of the effects of ATRA on the production of hematopoietic cell types from LKS+ are given in Figure 2.

The different effects of ATRA in hematopoiesis may be due to the cell target, the RAR(s) activated in such cells, or both. We and others have recently examined the expression of the different RARs in purified populations of murine hematopoietic cells and have found that the RARs are differentially expressed in different cell types [27, 28]. LKS+ cells (which contain HSCs and which have increased repopulating potential in response to ATRA) express RAR*α*1, RAR*α*2, RAR*β*2, RAR*γ*1, and RAR*γ*2 [27]. In contrast, LKS− cells (which do not contain HSCs and which differentiate in response to ATRA treatment) have similar RAR expression to LKS+ but do not express RAR*β*2 or RAR*γ*1 [27]. Additional data using RAR knockout mice have revealed distinct roles for the RARs in hematopoiesis.

### 1.6. Roles of retinoic acid receptors in hematopoiesis

Previous studies have investigated the role of pharmacological levels of ATRA in cultured hematopoietic cells. Such studies do not, however, provide insight of the physiological roles of the RARs in hematopoiesis. The importance of RAR*α* in granulopoiesis is demonstrated in APML patients, whose leukemic cells have aberrant chromosomal translocations that result in fusion of the RAR*α* gene with other genes, such as PML and PLZF [29]. These fusion gene products ultimately result in a block in promyelocytic differentiation, resulting in leukemia. Additional support for physiological roles of RARs in hematopoiesis comes from studies of mice either given a vitamin-A-deficient diet [30] or fed with a pan-RAR antagonist [31], who exhibit a dramatic increase in myeloid cells in bone marrow, spleen, and peripheral blood. However, while this underscores the importance of RARs in hematopoiesis, it does not discriminate between roles of each of the different RAR subtypes in hematopoiesis. ATRA, the most widely used retinoid in therapeutic applications at present, activates all three RAR subtypes. Each of the three RARs were previously considered to have similar effects in different organs, however recent data using mouse models or RAR-specific ligands are now emerging to challenge and even disprove this concept. Some studies on RAR knockouts have also begun to delineate the different roles of the RARs in hematopoiesis, and are discussed below.

### 1.7. Studies of HSCs in RAR-knockout mice

Mice null for RAR*α*, RAR*β*, or RAR*γ* all survive birth, but both RAR*α*- and RAR*γ* knockout mice exhibit early lethality [32–34]. Subsequent double null mice generated from these RAR subtype null mice have more profound defects, and die at the latest by 12 hours after caesarean delivery at E18.5 [35]. The triple null mouse has not been reported to date.

Previous reports on hematopoiesis in RAR null mice have been two separate studies on granulocyte development in RAR*α*1 and full RAR*α* knockouts. Both demonstrated that RAR*α* is not an important physiological regulator of granulocytes [28, 36]. Mice lacking both RAR*α*1 and RAR*γ* did exhibit a block in in vitro terminal differentiation into granulocytes, but this was not observed in vivo, suggesting that in vivo compensatory mechanisms in these double null mice restore normal granulopoiesis [28].

Both RAR*α* and RAR*γ* are the most widely expressed in hematopoiesis, including HSCs, hence we have investigated the HSC content in 8-week-old RAR*α* and RAR*γ* null mice. The RAR*α* null mice had normal HSC content, as assessed by limiting dilution analysis [27]. In contrast, whole bone marrow obtained from RAR*γ* null mice had a 3.3-fold reduction in the number of long-term repopulating HSCs in primary transplant recipients compared to that of their wild-type littermates [27]. Interestingly, bone marrow from RAR*γ* heterozygous mice had 2-fold fewer HSCs than the wild-type littermates, further highlighting the importance of RAR*γ* signalling in the regulation of HSCs [27]. The reduced numbers of HSCs observed in RAR*γ* null bone marrow was accompanied by increased numbers of more mature progenitor cells (CFU-S and CFCs), suggesting that RAR*γ* is critical for maintaining a balance between HSC self-renewal and differentiation [27]. 

The response of enriched populations of HSCs (LKS+) obtained from RAR mutants to ATRA treatment was also monitored in *ex vivo* cultures. HSCs obtained from RAR*α* null mice retained a normal response to ATRA treatment, as measured by prolonged and enhanced cell proliferation and their ability to reconstitute mice after 14 days of *ex vivo* culture [27]. In contrast, ATRA-treated LKS+ isolated from RAR*γ* null mice had markedly impaired proliferation and did not reconstitute mice after 14 days of culture [27]. Collectively, these studies demonstrate that ATRA-induced HSC self-renewal requires RAR*γ* signalling.

RAR*γ* has therefore been identified as being a key regulator of HSC self-renewal: activation of RAR*γ* enhances self-renewal, whereas inactivation of RAR*γ* enhances HSC differentiation, resulting in increased numbers of more mature progenitor cells. 

The recent generation of RAR-specific ligands [37] has made future studies of the effects of gain of function of different RARs on hematopoietic cells possible, and will likely lead to further therapeutic applications for retinoids in hematopoiesis.

### 1.8. Regulators of retinoid signaling: aldehyde dehydrogenase family

Little is known about the regulators of RARs in organogenesis. One major way of regulating activity of the RARs is by altering the availability of the biologically active retinoic acid ligands. A series of sequential enzymes with different specificities regulate the production of retinoic acid from retinol [7]. The important enzymes involved in the NAD-dependent oxidation of the aldehyde forms of vitamin A into ATRA and 9-*cis* retinoic acid are those of the aldehyde dehydrogenase (ALDH) family.

Like RARs, the ALDHs are highly conserved amongst vertebrates. There are numerous members of this family, not all of which can use retinoids as substrates. The cytosolic class 1 enzymes, retinaldehyde dehydrogenase 1 (RALDH1), RALDH2, and RALDH3, are the ALDH forms important for the conversion of retinal into retinoic acid forms [7]. All three enzymes are expressed differentially in embryogenesis and throughout later mouse organogenesis [38].

RALDH1 (also known as ALDH1, ALDH1A1, RalDH1, and Ahd2) is expressed in both embryonic and adult tissues and is capable of converting both all-*trans* retinal and 9-*cis* retinal into their respective carboxylic acid forms, hence providing ligands for both the RARs and RXRs [7]. *Raldh1* knockout mice are viable, with no apparent defects in growth or survival [39]. RALDH2 (also known as ALDH1A2) is more important embryonically, and *Raldh2* knockout mice die by E10.5, exhibiting multiple defects and a block in embryonic retinoic acid synthesis [40]. Interestingly, this lethal phenotype can almost be completely overcome by maternal retinoic acid administration, demonstrating that the defects in these mice are predominantly due to lack of retinoic acid. RALDH3 (also known as ALDH1A3 and ALDH6) is expressed in the ventral retina in the developing eye, olfactory regions, and other organs [38, 41]. *Raldh3* knockout mice are born, but die from respiratory distress within 10 hours of birth [42]. To date there have been no reports on hematopoiesis in any of the *Raldh* mutants.

A series of recent reports have shown that both murine and human primitive HSCs and progenitors are contained within the lineage-negative, ALDH high fraction, and can be isolated based on ALDH activity [43–47]. In contrast, the population of murine hematopoietic cells lacking ALDH1 expression did not contain HSCs [44]. These data therefore not only reinforce the importance of RAR signalling in HSCs, as shown in our recent studies, but also provide evidence that HSCs themselves are capable of generating ATRA and 9-*cis* retinoic acid from retinal. A summary of the expression of RARs and ALDH1 in murine HSCs and progenitor cells is given in Table 1.

One study to date has reported that inhibiting ALDH1 in human hematopoietic stem/progenitor cells in vitro induces their expansion and prevents their differentiation [48]. Further studies of the roles of the aldehyde dehydrogenases in the regulation of HSCs are therefore of interest.

### 1.9. Therapeutic applications of retinoids for HSCs

Given that the retinoid pathway is highly conserved between human and mouse, it is now of interest to determine whether ATRA has the same effects on human HSCs. Some obstacles to these translational studies are that (1) the population enriched for human HSCs is much more heterogeneous than the one that can be obtained for murine HSCs, which presents potential problems given the pleiotropic effects of ATRA and (2) the NOD/SCID mouse repopulating assay, which to date is the best small animal model for in vivo transplantation studies of human HSCs, may not be reflecting true HSC activity of the cell population [49]. Nevertheless, a recent report demonstrated that ATRA could support the expansion of SCID-repopulating cells (SRC), human hematopoietic cells that are capable of repopulating NOD/SCID mice [50]. These effects of ATRA on human hematopoietic stem/progenitor cells relied on the presence of a stromal feeder layer, but did not require contact between the stromal cells and HSPCs [50]. It is therefore likely that ATRA induced the secretion of substances from the stromal cells that were capable of expanding HSPC. The potential use of retinoids to expand human HSCs for therapeutic purposes therefore warrants further investigation: in particular, given its profound roles in murine HSCs, it is of interest to determine the effects of specifically activating RAR*γ* in these cells.

## 2. CONCLUSION

It is becoming apparent that the roles of retinoids and their receptors in hematopoiesis are complex, having pleiotropic effects depending on the hematopoietic target cell. In contrast to its potent differentiation-inducing effects on granulocyte progenitor cells, ATRA enhanced the self-renewal of HSCs. These different effects are likely due to the effects of the distinct RARs in hematopoiesis. RAR*α* has a clear role in enhancing granulocyte maturation, as demonstrated by both its involvement in APML [29] and also the potent effects of an RAR*α*-specific ligand on granulocyte differentiation [22]. We also recently reported that an RAR*α*-specific ligand enhanced the mobilization of murine hematopoietic stem and progenitor cells into the peripheral blood for transplantation purposes via increasing the numbers of immature granulocyte progenitors in vivo [51]. Interestingly, these effects were not seen when ATRA was used in place of the RAR*α*-specific ligand, perhaps due to contrasting effects obtained by activating all three different RARs concurrently, a possibility that further adds to the complexity of the effects of retinoids in hematopoiesis. In contrast, RAR*γ* is a major regulator of HSC self-renewal: gain of function of RAR*γ* enhances HSC self-renewal, whereas loss of function of RAR*γ* promotes differentiation of HSCs [27]. These distinct effects of the RARs in hematopoiesis suggests that, in the future, therapeutically targeting the RARs via RAR-specific ligands may have a more profound effect on the target cell than by using the pan-RAR agonist ATRA. Such studies will also permit further delineation of the roles of the RARs in HSC biology.

## Figures and Tables

**Table 1:** Expression of RARs and ALDH1 in murine HSCs and progenitor cells. Positive expression is indicated by (+) and negative expression by (−). Summary of data is obtained from references [27] and [44].

	Hematopoietic cell population	
	HSCs	Progenitors

RAR*α*1	+	+
RAR*α*2	+	+
RAR*β*2	+	−
RAR*γ*1	+	−
RAR*γ*2	+	+
ALDH1	+	−
